# Analysis of human ABO Blood Group as a risk factor with knee osteoarthritis at tertiary care hospital in Pakistan

**DOI:** 10.12669/pjms.40.5.8865

**Published:** 2024

**Authors:** Shazia Rahman Shaikh, Aneela Atta Ur Rahman, Asadullah Makhdoom

**Affiliations:** 1 Dr. Shazia Rahman Shaikh, MBBS, MPH. Department of Community Medicine and Public Health Sciences, Liaquat University of Medical and Health Sciences (LUMHS), Jamshoro, Sindh, Pakistan; 2 Prof. Dr. Aneela Atta Ur Rahman, MBBS, MPH, PhD. Department of Community Medicine and Public Health Sciences, Liaquat University of Medical and Health Sciences (LUMHS), Jamshoro, Sindh, Pakistan; 3 Prof. Dr. Asadullah Makhdoom, MBBS, MS. Department of Orthopaedic Surgery and Traumatology, Liaquat University of Medical and Health Sciences (LUMHS), Jamshoro, Sindh, Pakistan

**Keywords:** KEYWORD: Knee Osteoarthritis, ABO Blood Group, Rh Antigen, Kellgren and Lawrence score

## Abstract

**Objective:**

To investigate the association and risk estimation of ABO blood group distribution and clinical attributes in patients with Knee Osteoarthritis.

**Method:**

This was a hospital-based study conducted at, Liaquat University Hospital Hyderabad from December, 2019 to December, 2022 to investigate this least researched area of highly prevalent musculoskeletal disease in Pakistan. Non-Probability Convenience Sampling was used for selecting 190 cases of confirm Knee Osteoarthritis patients diagnosed by Orthopedic surgeon based on standard clinical and radiographic criteria. Data were analyzed using IBM-SPSS version 23.0. Percentages and frequencies were counted for categorical data. Pearson Chi Square test and fisher’s exact test were used to check the association and Multinomial Logistic Regression was used to estimate the risk for moderate and severe kellgren grading Knee Osteoarthritis (KOA) cases with ABO blood grouping in comparison of mild Kellgren grading.

**Results:**

A total of 190 cases of Knee Osteoarthritis (KOA) were included in the study. Females (61.6%) and patients with age 50 and above were 40.5 % were found in greater proportion. Majority (41.6%) were classified radiologically as mild cases with O group (39.5%) and positive Rh antigen (95.8%). Strong association (p = <0.01) was found between gender, age group and ABO blood group with KOA radiological Kellgren and Lawrence score.

**Conclusion:**

There is strong relation in between radiological grading of knee osteoarthritis severity and A blood group, gender and age.

## INTRODUCTION

An estimated 300 million people worldwide suffer with osteoarthritis (OA), which is one of the most prevalent types of arthritis that affects the joints. Over the past 20 years, the prevalence of symptomatic osteoarthritis and knee pain has almost doubled in women and tripled in men. It is an inflammatory, degenerative and multifactorial disorder of the joints leading to disability.^1^-^3^ Recent researches indicates that the inflammatory process plays a significant role in the pathogenesis of OA. It is suggested that there is a link between inflammatory cytokines and knee OA, Latest research reveal that the process of inflammation has a role in the course of OA. It has been hypothesized that there is a link between inflammatory cytokines and progression of knee OA, because they cause the cartilage, synovial tissue, subchondral bone and synovial fluid, to interact, speeding up the degradation of articular cartilage.^4^ Additionally, research has demonstrated that the complex process of OA comprises an interaction between ABO blood group, an inflammatory response, and metabolic disorders.^3^

The ABO blood group system was invented in 1901 by Austrian pathologist Karl Landsteiner after he detected variations in agglutination during blood transfusions and labelled them A, B, AB, or O blood groups, on the basis of the presence or absence of A and B antigens on the surface of red blood cells (RBCs). ABO groups on the basis of Rh system were further divided into Rh (-) and Rh (+) subgroups according to the presence of the Rh-D antigen on the surface of RBCs.^5^,^6^ ABO phenotypes and alleles are frequently the focus of epidemiological and anthropological investigations since they are genetically determined features which are mostly race-dependent, and there are significant differences among the racial populations as Caucasians, Asians, and Negros. Additionally, numerous studies on the relationship between diseases and blood types have demonstrated the importance of ABO blood types as hereditary risk factor for a number of human diseases.^6^ Scientists have studied the association between the ABO blood type and diseases since it was found in 1953 that having blood group A increased the likelihood of developing stomach cancer. Since then, blood group has been related as a risk factor to a variety of diseases.^4^

Infection susceptibility has been observed to be influenced by ABO blood types, supporting the idea that interactions between host and pathogen may be affected by the diversity of blood groups seen in the population.^7^ According to research, inflammation is considered as one of the major pathogenic factors influencing the relationship between primary knee osteoarthritis and the ABO blood group. In this study, the distribution of ABO and Rh blood types in patients with knee osteoarthritis was evaluated. Additionally, the relationship between primary knee OA and the ABO blood group was investigated, as well as its relationship to the degree of severity as determined by the radiological Kellgren and Lawrence (K/L) grading.

## METHODS

It was a hospital-based study carried out on patients with primary knee osteoarthritis treated at the Department of Orthopaedic Surgery & Traumatology outpatient clinic at Liaquat University Hospital Hyderabad between December, 2019 and December, 2022.

The study subjects were diagnosed by an expert orthopedic surgeon and classified on the basis of clinical^8^ and radiological^9^ criteria of knee osteoarthritis. The sample of 190 individuals with radiographic criteria for Kellgren and Lawrence (K/L) Grade-2 or more were investigated. This is the most frequently used radiological grading method for evaluating the severity of knee OA. These patients were then split into three groups on the basis of radiographic classification as Mild KOA for Grade-2, Moderate KOA for Grade-3, and Severe KOA for Grade-4.

The inclusion criteria for cases were age 30 years and above, and both genders, with knee pain or functional limitations during past month, whereas the patients, with trauma, congenital anomaly of lower limb or having any serious arthropathy as rheumatoid arthritis, ankylosing spondylitis were excluded. All participants who provided written consent were invited, and information was collected through a face-to-face interview session using a structured questionnaire.

### Ethical Approval:

This research was approved by the Ethics Review Committee of Liaquat University of Medical and Health Sciences, Jamshoro (Letter No. LUMHS/REC/713, Dated 29/08/2018).

### Statistical analysis:

Analysis of 190 diagnosed patients was done in concordance with ABO blood group distribution and with clinical traits of KOA. IBM-SPSS version 23.0 was used to store and analyze the data. Counts and percentages were presented for each age group, gender, blood type, Rh type, and KOA K/L Grading. The association of K/L grading with gender, age group, ABO blood and Rh group has been investigated using the Pearson Chi Square test and the fisher’s exact test. Multinomial Logistic Regression has been applied to assess the risk for moderate and severe K/L grading of KOA cases to mild K/L grading. Odds ratios were presented along with a 95% confidence interval. P-values less than 0.05 were used to evaluate statistical significance.

### Consent:

To participate in the study before getting the sign of an informed consent form, the eligible participants were briefed about study objectives.

## RESULTS

A total of 190 patients who came to the Out-Patient Department (OPD) of Medicine and Orthopedics with primary knee osteoarthritis were included in the study. All study participants were aged 30 and above, with a mean age of 40.5 ± 7.1 years and a clear female predominance (61.6%).

The descriptive statistics regarding the parameters that have been investigated in this study is summarized in Table-I. There were one hundred and ninety samples, out of that 61.6% were female gender, 31.6% with age 40 – 49 years old, Mean age was 40.5 (SD=±7.1) years, 41.6% were mild KOA, 39.5% with O blood group and 95.8% were Rh positive samples.

**Table-I:** Descriptive on Gender, Age group, Severity of Kellgren and Lawrence KOA Grading, Blood group and Rh Group (n=190).

Variables		n	%
Gender	Male	73	38.4
	Female	117	61.6
Age Group	30 – 39 years	53	27.9
	40 – 49 years	60	31.6
	≥50 years	77	40.5
	Mean ±SD	40.5	±7.1
Kellgren Grading	Mild KOA	79	41.6
	Moderate KOA	77	40.5
	Severe KOA	34	17.9
Blood Group	A	39	20.5
	B	64	33.7
	AB	12	6.3
	O	75	39.5
Rh Group	Rh Positive	182	95.8
	Rh Negative	8	4.2

The association of Kellgren and Lawrence KOA grading with studied parameters is reported in Table-II. In Mild KOA 50.6% were female gender, 24.1% were age fifty or above, 40.5% were with “B” blood group and 96.2% were Rh positive. In moderate KOA 75.3% were female gender, 37.7% were age fifty or above, 28.6% were with “B” blood group and 96.1% were Rh positive, whereas in severe KOA 55.9% were female gender, 85.3% were age fifty or above, 17.6% were with “B” blood group and 94.1% were Rh positive. Pearson Chi Square test did give significant association of K/L grading with gender and age group (p<0.01), and significant association was found for blood group using Fisher’s Exact test (p<0.01), no significant association was found for Rh with K/L gradings (p=0.17).

**Table-II:** Association of Kellgren and Lawrence KOA Grading with Gender, Age, Blood and Rh Group.

Variables		Kellgren and Lawrence KOA Grading						

		Mild KOA		Moderate KOA		Severe KOA		p-value
		*n*	%	*n*	%	*n*	%
Gender	Male	39	49.4	19	24.7	15	44.1	<0.01*
	Female	40	50.6	58	75.3	19	55.9	
Age Group	30-39 years	30	38.0	21	27.3	2	5.9	<0.01*
	40-49 years	30	38.0	27	35.1	3	8.8	
	≥50 years	19	24.1	29	37.7	29	85.3	
Blood Group¥	A	12	15.2	27	35.1	0	0.0	<0.01*
	B	32	40.5	22	28.6	10	29.4	
	AB	5	6.3	1	1.3	6	17.6	
	O	30	38.0	27	35.1	18	52.9	
Rh Group¥	Rh Positive	76	96.2	74	96.1	32	94.1	0.17
	Rh Negative	3	3.8	3	3.9	2	5.9	

* p<0.05 was considered statistically significant using Pearson Chi Square test ¥: Association was tested using Fisher’s Exact test.

The risk estimation of Kellgren and Lawrence KOA grading with studied parameters using Multinomial Logistic Regression Analysis. Is shown in Table-III. For Moderate KOA male gender gives significant association [OR = 2.97 CI (1.50 – 5.87)], blood group “A” also gives significant positive association [OR = 2.5 CI (1.06 – 5.88)]. For Severe KOA cases samples with age group 30 – 39 years shows significant negative association [OR=0.04 C.I (0.01 – 0.20)], and also age group 40 – 49 indicates significant negative association [OR=0.06 C.I (0.01 – 0.24)]. Association of Rh-positive cases with K/L grading was negative, however it was not statistically significant.

**Table-III:** Risk Estimation of Kellgren and Lawrence KOA Grading using Multinomial Logistic Regression.

Factors	Moderate KOA Odds Ratio 95% C.I	Severe KOA Odds Ratio 95% C.I
Male	2.97* (1.50-5.87)	1.23 (0.55-2.77)
Age Group 30 – 39 years	0.45(0.20-1.02)	0.04* (0.01-0.20)
Age Group 40 – 49 years	0.58(0.27-1.28)	0.06* (0.01-0.24)
Blood Group A	2.5*(1.06-5.88)	N.A
Blood Group B	0.76(0.36-1.61)	0.52(0.20-1.30)
Blood Group AB	0.22(0.02-2.02)	2(0.53-7.50)
Rh Positive	0.97(0.19-4.97)	0.63(0.10-3.96)

* Odds ratio considered statistically significant with p<0.05.

## DISCUSSION

The main components of the ABO blood group system are the A and B antigens and related antibodies. There are four genetic phenotypes (blood types A, B, O, and AB) made up of the A, B, and O alleles. Host vulnerability to numerous illnesses can be increased or decreased by variations in blood group antigen expression. Blood group antigens can act as receptors in an infection, contributing to it directly. Blood-borne innate immune antibodies can be altered by blood group antigens. There might be other mechanisms, though, that need more research.^10^

ABO blood grouping are quick and quiet inexpensive tests that can be obtained from patients for the purpose of analyzing the relationship between variables of interest. The current investigation examined the link between blood grouping and the severity of primary knee OA as determined by the Kellgren/Lawrence (K/L) grading system, the most widely used radiological grading system for assessing disease severity. The strength of this classification system and diagnostic criteria used in the study is a recognized way to confirm the patients with KOA.

The mean age of KOA patients in the current study was 40.5±7.1, which is supported by another study conducted on rheumatic diseases in India (45.56 ±13.08 years).^11^ Also Yaradilmis et al. 2021 in their study reported mean age 67.2 +/- 8 years, which supports the disease an aging risk factor.^12^ In our study, we found that women are more likely than men to develop knee osteoarthritis, and this was reported with a higher percentage (61.6%), which is consistent with the results of other studies that also support the female gender as a non-modifiable etiological risk factor of the condition.^1^,^12^,^13^

The ABO blood group has four blood types (A, B, AB, and O) and two antigens (A and B antigens). The ABO gene produces the A and B antigens, which are autosomal dominant. An autosomal-recessive trait is the group O phenotype. A, B, and H denote the antigens, while ABO denotes the blood type. Although group O tends to be the most prevalent ABO type, the proportional distribution can fluctuate between ethnic groupings.^14^ The expression of antigen A and B determines the blood group. Each person exhibits different degrees of H antigen and blood group O individuals exhibit higher levels of H antigen expression, whereas blood group AB persons exhibit lower levels. Few of the previous studies suggested that blood group O would be a protective factor,^4^ but the extracted information from KOA patients of current study shows over-representation of blood group O (39.5%), and around same is seen in study conducted by Li C 2020 with (38.4%) with O blood group.^4^ But the study on Turkish population showed strong correlation between group A and osteoarthritis (p=0.001), yet an important but weak association existed between group AB (p=0.002).^12^ Though group AB is found least (6.3%) in the subjects of present study.

The results of our study shows more individuals with Rhesus (Rh) (+) factor (95.8%) than Rh (-) (4.2%) which is not different from the study conducted by other researcher.^11^ Another regional study undertaken on a larger sample of 10,000 with a view to provide information on the frequency distribution of ABO and Rh(D) blood groups within their population demonstrates Rh positive in 94.61% of people, whereas Rh negativity in the remaining people.^15^ This supports that Rh +ve factor is prevalent more in the population as compared to the other one.

Osteoarthritis (OA) is a degenerative joint disease with multiple pathophysiological factors, which affects the synovium, articular cartilage, subchondral bone, and joint capsule. The predominant pathologic finding of OA is cartilage degeneration; however, the pathophysiology of OA also involves inflammation of the synovium (synovitis). Synovitis frequently develops prior to structural damage to the joints, and its presence is linked to the advancement of OA.^16^ Tumor Necrosis Factor (TNF) and Inter-Cellular Adhesion Molecule 1 (ICAM-1), which are significant pro-inflammatory cytokines, affects the systemic inflammatory response and have recently been linked to the ABO gene by genome-wide research. It is also well known that the pathophysiology and development of primary knee OA are strongly influenced by an unbalanced level of pro-inflammatory cytokines and a disorganized inflammatory process.^4^ Therefore, association of ABO blood group and radiographic severity of KOA was assessed which showed statistically significance with p = <0.01 in current study, whereas Li et al 2019 in his research found no any significant difference (*P* = 0.124) when compared the K/L score of patients with blood groups.^4^ Results of the current study revealed risk estimation of blood grouping with K/L OA severity grading using Multinomial Logistic Regression and blood group A was found as risk factor associated with the severity of knee osteoarthritis (2.5, CI=1.06-5.88) whereas in the study of Li AB group was identified a risk factor.^4^ Several investigations were undertaken during the COVID pandemic to examine how the ABO blood group relates to the susceptibility of SARS-CoV-2 infections. One of them from China revealed that blood group O was linked to a lower risk of SARS-CoV-2 infection while blood group A was associated with an increased risk.^10^ A study from India on rheumatic diseases revealed that in comparison to people with O- ve blood group, patients with blood types A+ve (0.98, CI=0.62, 1.54, P=0.0037) and O+ve (1.08, CI=0.694, 1.68, P=0.0215) had a lower risk of developing rheumatic diseases.^11^

To the best of our knowledge, very little data was available, particularly from Pakistan, hence it would be suggested that large-scale investigations with different study designs might be carried out at local and national level with the aim to address the research gap.

### Limitations:

The study has some limitations, including an inadequate number of study participants and the lack of a comparison group, which limits the generalizability of the findings that can be applied. However, it can serve as a baseline for studies that will be conducted in the future to gain a better understanding.

## CONCLUSION

Conclusions from our study indicate a link between blood group type and illness severity. Gender and advanced age as etiological factor are particularly affected by the rising prevalence of arthritis. As a result, it is hypothesized that a blood grouping test, which is frequently inexpensive and simple to conduct, may be used as a useful indicator to foresee the outcome so as to take preventive measures at the appropriate time.
